# Effects of maslinic acid supplementation on exercise-induced inflammation and oxidative stress in water polo athletes: A randomized, double-blind, crossover, and placebo-controlled trial

**DOI:** 10.1080/15502783.2023.2239196

**Published:** 2023-07-27

**Authors:** Takanaga Shirai, Kanae Myoenzono, Eiskue Kawai, Yuki Yamauchi, Keito Suzuki, Seiji Maeda, Hideki Takagi, Tohru Takemasa

**Affiliations:** a University of Tsukuba, Faculty of Health and Sport Sciences, Tsukuba, Ibaraki, Japan; b Research Fellow of the Japan Society for the Promotion of Sciences, Chiyoda-Ku, Tokyo, Japan; c Japan Institute of Sports Sciences, Kita-Ku, Tokyo, Japan; d International Budo University, Faculty of Physical Education, Katsuura, Chiba, Japan; e University of Tsukuba, Tsukuba Life Science Innovation Program (T-LSI), Tsukuba, Ibaraki, Japan; f Nippn Corporation, Central Research Laboratory Innovation Center, Atsugi, Kanagawa, Japan; g Waseda University, Faculty of Sport Sciences, Tokosozawa, Japan

**Keywords:** Maslinic acid, water polo athletes, fatigue, muscle soreness, inflammation

## Abstract

**Background:**

Olive fruit is rich in bioactive pentacyclic triterpenoids, primarily maslinic acid (MA). Previous studies have demonstrated that MA exhibits anti-inflammatory and anti-oxidative effects; however, it is unclear whether MA intake during training inhibits perceptual fatigue and muscle soreness in athletes. This study analyzed the effects of MA supplementation during athletic training on perceptual fatigue and muscle soreness.

**Methods:**

This randomized, double-blind, cross-over, and placebo-controlled trial involved 12 young, healthy male water polo athletes. After daily training for seven days, they ingested either olive fruit extract, containing 60 mg/day MA, or a placebo. We measured perceptual fatigue and muscle soreness during the intervention using a visual analog scale and inflammatory and oxidative stress-related proteins.

**Results:**

Perceptual fatigue and muscle soreness and the area under the curve during the training period were significantly lower (main effect of MA; *P* < 0.05) following MA supplementation than those for the placebo. MA supplementation during training lowered perceptual fatigue and muscle soreness by decreasing inflammatory factors in water polo athletes. Additionally, we examined the detailed mechanism of MA, added the participant’s serum to the culture medium at a 10% concentration to determine inflammation- and oxidative stress-related intracellular signals. Skeletal muscle cells (C2C12) cultured with MA-conditioned serum before and after intervention also suppressed expression of inflammation and oxidative stress-related proteins.

**Conclusion:**

These findings suggest that MA intake not only reduces perceptual fatigue and muscle soreness but also decreases inflammation and oxidative stress in the blood and skeletal muscle.

## GRAPHICAL ABSTRACT

**Figure:**
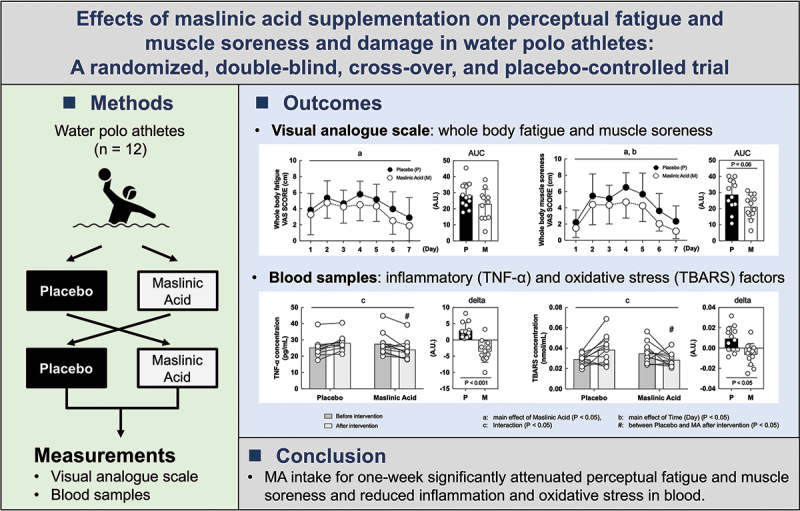

## 1. Introduction

Chronic fatigue can impact physical and mental athletic performance. A tired athlete may have less energy to exert themselves during practice or a game. Exercise-induced fatigue, which is attributable to many factors, including the accumulation of reactive oxygen species, inflammation, and muscle glycogen depletion, affects athletic performance [1–4]. However, fatigue and the mechanism leading to the perception of fatigue are not fully elucidated. Empirically, rest, sleep, bathing, nutrition, light exercise, and aromatherapy are commonly used to recover from fatigue [5–7]. In particular, to prevent exercise-induced fatigue, consuming sufficient carbohydrates and amino acids before exercise to store energy is widely practiced [8,9]. An additional crucial nutritional strategy to lessen fatigue, enhance energy usage, recover faster from exercise, and has become more prevalent in many sporting situations is the use of antioxidant and anti-inflammatory component through diet and supplements [10]. Therefore, it is necessary to identify food ingredients that can be readily taken.

Olives are an essential oilseed crop that has been consumed since ancient times. Recent studies have indicated that maslinic acid (MA) with pentacyclic triterpene structures from olive extracts have various health benefits [11–14]. In previous animals and human studies, MA reportedly had a robust anti-inflammatory effect [13]. It suppressed knee joint inflammation in arthritis-induced mice [12] and reduced fibrosis, such as fatty liver, in animal models [15]. MA effectively reduces fatty liver and oxidative stress in mice [16,17] and improved kidney function in diabetes mellitus patients [18]. MA intake exhibits beneficial effects on the whole-body, including suppressing high-sensitivity C-reactive protein (hs-CRP) in the blood and reducing perceptual pain in elderly humans [13]. We have reported that MA promotes the muscle hypertrophic response by activating mechanistic target of rapamycin (mTOR) both *in vivo* and *in vitro* [19,20]. These reports indicate that MA is an effective supplement for the health of individuals.

Water polo is a goal-based ball game played in the water, and it consists of such a robust offense and defense that it is considered “underwater fighting” because of its intensity. Water polo is characterized by complex activities, such as swimming with various strengths, and treading water [21–23], contacting with opponents, passing the ball to teammates, and goal shooting [24]. Therefore, water polo athletes utilize training and nutrition strategies to optimize these skills to improve their competitive performance [25,26].

Most athletic performance studies focus on conditioning, such as peaking and tapering [27,28]. In particular, water polo athletes require a higher level of whole-body endurance, muscular strength, and power as they play in high resistance of water with intense contact [22,29,30]. In this sport, the athletes are also exposed to external stresses, such as water temperature, which is expected to increase the fatigue accumulation [31–34]. Athletes who engage in high-intensity training every day need to recover quickly to avoid fatigue accumulation and to perform each training session efficiently [35]. Despite the aforementioned benefits of MA supplementation, its effects on performance, inflammation, and oxidative stress in water polo athletes are unknown. Additionally, the effects of oleanolic triterpenes, such as MA, on fatigue and muscle soreness in athletes who perform daily high-intensity training have not been examined. Based on previous studies, we hypothesize that MA intake in water polo athletes suppresses fatigue and muscle soreness through its antioxidant and anti-inflammatory effects. Therefore, we determined the effects of MA intake on perceptual fatigue and muscle soreness levels in water polo athletes during training.

## 2. Materials and methods

### 2.1. Participants

This randomized, placebo-controlled, double-blind trial was conducted with 12 Japanese first-division national-level male university water polo athletes. The characteristics of the participants are shown in Table 1. The participants had five water polo training sessions per week during this study (Table 2). All measurements, except those of daily physical activity, were obtained in the morning after overnight fasting for approximately 10–12 h, during which water intake was allowed, but abstaining from caffeine, alcohol, and medication was required. In addition, the participants were instructed to avoid exercise for at least 24 h before the measurements. Each participant received an oral explanation of the potential risks and benefits of the study and provided written informed consent. The University of Tsukuba Research Ethics Committee approved this study (Tai29–75), which complied with the principles of the Declaration of Helsinki. This study was registered in the UMIN Clinical Trial Registry under the ID UMIN 000030479.

**Table 1.** Grouping condition and characteristics of participants.

Condition	Placebo		Maslinic Acid	
	Before intervention	After intervention	Before intervention	After intervention
Age (years)		19.9±0.9		
Height (cm)		177.2±7.6		
Body weight (kg)	76.3 ± 12.5	75.5 ± 11.7	75.7 ± 11.9	75.9 ± 11.9
BMI (kg/m^2^）	24.2 ± 3.0	24.1 ± 2.9	24.1 ± 2.9	24.3 ± 3.0
Muscle mass (kg)	36.0 ± 4.2	36.1 ± 4.6	36.0 ± 4.4	36.1 ± 4.6
Fat (%)	16.2 ± 5.5	15.0 ± 5.6	15.9 ± 5.2	15.9 ± 5.0

**Table 2.** Training schedule from Day 1 to Day 7 during intervention.

		Day 1	Day 2	Day 3	Day 4	Day 5	Day 6	Day 7
AM	Swim	Swim set (1200 m)	Swim set (1200 m)	Swim set (1200 m)	Swim set (1200 m)	Swim set (1200 m)	Game play	Testing
		Swim drills	Swim drills	Swim drills	Swim drills	Swim drills		
		Medicine ball throwing	Medicine ball throwing	Medicine ball throwing	Medicine ball throwing	Medicine ball throwing		
		tube pull	tube pull	tube pull	tube pull	tube pull		
PM	Skill	Passing	Passing	N.A.	Passing	Passing	N.A.	
		Shooting	Shooting		Shooting	Shooting		
		One side game	One side game		One side game	One side game		
		Match simulation	Match simulation		Match simulation	Match simulation		
	Training	N.A.	Bench press	N.A.	Bentover row	N.A.	N.A.	
			Side raise		Chin up			
			Shoulder press		Dead lift			
					Arm curl			
S: Sets, R: Repetitions, RM: Repetition Maximum, BW: Body Weight								
Bench press		6S × 10 R at 70 ~ 85% 1RM						
Side raise		4S × 15 R at 20 kg						
Shoulder press		6S × 10 R at 30 ~ 40 kg						
Bentover row		6S × 10 R at 70 ~ 85% 1RM						
Chin up		6S × 10 R at BW						
Dead lift		4S × 10 R at 70 ~ 85% 1RM						
Arm curl		6S × 10 R at ~30 kg						
※Training excludes w-up.								

### 2.2. Study design

**Figure 1.**
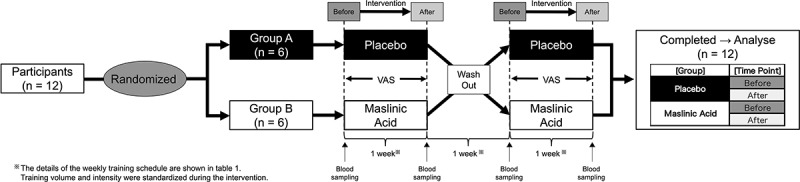
Study design.

Figure 1 outlines the experimental protocol. The present study was a randomized, double-blind, cross-over, and placebo-controlled trial. MA or placebo was administered for 1 week followed by a 1-week washout period. All measurements were taken before and after the intervention. The participants were instructed to avoid alcohol, caffeine, and strenuous exercise on the day before the measurements, and all participants assembled at our laboratory at 07:00 after fasting overnight. After anthropometric measurements and blood sampling, a performance trial (eggbeater kick) was conducted. After training during their routine practice, the participants promptly ingested daily jelly containing 60 mg of MA derived from olive fruit (NIPPN CORPORATION, Tokyo, Japan) or placebo (without MA). Both experimental supplements included an equivalent amount of water, gelling agent, pH adjusters, sweetener, and aroma chemicals. However, the placebo jelly contained dextrin and young leaves instead of olive extract, although all test diets were visually identical. This was done continuously for seven days. The participants maintained their routine of intense physical and technical training. The training program consisted of integrated exercises to improve strength, speed, power, agility, and endurance capacity for swimming and specific training for water polo.

### 2.3. Training schedule

The participants maintained a fixed training regimen (Table 1). All training (technical skill, weights, and swimming) performed during the 7-days intervention was identical and took place in the controlled environment of the indoor water polo pool or gymnasium.

### 2.4. Anthropometric measurements

Anthropometric measurements were performed bare feet in light clothes. Body mass was measured to the nearest 0.1 kg using a digital scale (InBody 770; InBody Japan, Tokyo, Japan). Height was measured to the nearest 0.1 cm using a wall-mounted stadiometer (Digital Height Meter AD-6227 by A&D, Tokyo, Japan). Body mass index (BMI) values were calculated by dividing the weight (kg) by the height (cm) square. Total body fat was estimated using bioelectrical impedance analysis (InBody 770).

### 2.5. Performance trial (eggbeater kick ability)

Participants conducted the performance trial measurements in a 50-m indoor pool with a maximum depth of 3.8 m. A blood sample was taken from the fingertip and blood lactate levels were measured with Lactate Pro2 (Arkray. Kyoto. Japan) before and after the performance trial. Participants performed a 20-second eggbeater kick while holding a 10-kg weight (with no arm sculls) and keeping their manubrium (superior part of the sternum) above the water surface. Afterward, the participants stood on the underwater sidewalk (staging point on the pool wall) and rested for 20 seconds with the weight on the poolside. This interval exercise (20-second eggbeater kick + 20-second rest) was performed as one set until exhaustion. During the test, subjects were instructed to maintain as high a body position as possible; when participants could no longer maintain the reference line (upper sternal border at the water surface), they were considered exhausted, and the test was stopped.

### 2.6. Perceptual assessments of fatigue and muscle soreness

The perceptual assessment of fatigue and muscle soreness in the whole or parts of body were surveyed using the visual analog scale (VAS) [36,37]. It consisted of a 100-mm line with a “no pain” or “no tired” description at one end and an “extreme pain” or “extreme tired” description at the other end. The VAS score was self-checked immediately upon waking up for 7 days. To avoid inter-measurer errors, the same measurer performed all VAS measurements. With reference to Tokinoya et al., values for fatigue or muscle soreness were expressed as the area under the curve (AUC) of values plotted from day 1 to day 7 of the intervention and calculated as the sum of trapezoidal areas separated at each time point [38].

### 2.7. Serum tumor necrosis factor α (TNF-α) levels and other biochemical markers

Blood samples were collected from each participant in the morning after fasting for 12 h overnight. Each blood sample was placed in a serum separator tube and centrifuged at 2,000 rpm for 15 minutes at 4°C. The serum was stored at −80°C until further analysis. Serum TNF-α levels were measured by ELISA using the Human TNF-α Assay kit (#KHC3011, Invitrogen – Thermo Fisher scientific, Tokyo, Japan), according to the manufacturer's protocol. Serum levels creatine kinase (CK), hs-CRP, and creatinine were determined using standard enzymatic techniques. These analyses were outsourced to Tsukuba i-Laboratory LLP (Tsukuba, Japan).

### 2.8. Thiobarbituric acid reactive substance (TBARS) assay

Serum TBARS were measured as described previously [39]. Briefly, 50 mg sample, 50 μL distilled water, and 950 μL master mix (0.2% w/v sodium dodecyl sulfate, 0.25% w/v thiobarbituric acid, butylated hydroxytoluene solution, acetic acid buffer 0.5% v/v acetic acid, 0.07% w/v sodium acetate) were mixed and incubated at 4°C for 1 h, followed by heating at 100°C for 1 h. The mixture was extracted with 1.0 mL of 1-butanol – pyridine (15:1, v/v), and the extract fluorescence was measured at an excitation of 532 nm and an emission of 585 nm.

### 2.9. Cell culture

Murine C2C12 skeletal muscle cells (passage numbers 10–12) were seeded into 12-well plates and maintained in Dulbecco’s modified Eagle’s medium (DMEM) (11966025, Gibco) containing 10% (vol/vol) fetal bovine serum (FBS; F9665, Sigma-Aldrich, St. Louis, MO), 1% (vol/vol) penicillin-streptomycin (15070–063, Gibco), 5 mM glucose (G7021, Sigma−Aldrich), 1 mM sodium pyruvate, and 1 mM GlutaMAX (35050–038, Gibco) under standard conditions (5% CO2, 100% humidity, 37℃). When the cells reached 90% confluence, the medium was switched to a differentiation medium consisting of DMEM supplemented with 2% calf serum (Bio West) and 1% nonessential amino acids (Invitrogen). This timing was designed Day 0. The differentiation medium was changed every 24 h. On Day 5 of differentiation, myotubes were changed to an amino acid and serum-free medium (D9800–13, US Biological, Salem, MA) (pH 7.3). The cells were incubated for 1 h without serum and amino acids prior to treatment. Myotubes were subsequently treated with a medium containing 10% human serum with placebo or MA before or after intervention for 24 h. The serum dose was determined from reflating previous studies, which ranged from 5% to 20% [40,41]. Therefore, given the limited serum sample availability and the results from preliminary experiments, a dilution of 10% was assigned optimal for the current experimental experiment.

### 2.10. Western blot analysis

Total protein from the C2C12 cells was extracted with a lysis buffer containing 50 mM HEPES (pH: 7.6), 150 mM NaCl, 10 mM EDTA, 10 mM Na_4_P_2_O_7_, 10 mM NaF, 2 mM Na_3_VO_4_, 1% (vol/vol) NP-40, 1% (vol/vol) Na-deoxycholate, 0.2% (wt/vol) SDS, and 1% (vol/vol) complete protease inhibitor cocktail (Nacalai Tesque Inc. Kyoto, Japan). Protein concentrations were measured using a Protein Assay Bicinchoninate Kit (Nacalai Tesque Inc. Kyoto, Japan). Before SDS-PAGE, an aliquot of the extracted protein solution was mixed with an equal volume of the sample loading buffer containing 1% (vol/vol) 2-mercaptoethanol, 4% (wt/vol) SDS, 125 mM of Tris-HCl (pH: 6.8), 10% (wt/vol) sucrose, and 0.01% (wt/vol) bromophenol blue. The mixture was then heated to 37℃ for 30 min. Then, 10 mg of protein was separated on SDS-polyacrylamide gels and electrically transferred to an ImmunoBlot PVDF membrane (Bio-Rad Laboratories, Hercules, CA, USA). The blot was blocked with the Blocking One (Nakalai Tesque Inc. Kyoto, Japan) for 1 h at room temperature and incubated with primary antibodies overnight at 4℃ in TBS containing 0.1% Tween-20. The signals were detected using the Immunostar Zeta or LD (Wako Chemicals. Osaka, Japan), quantified using the C-Digit (LI-COR Biosciences, Lincoln, NE, USA), and expressed as arbitrary units.

### 2.11. Primary antibodies

The following primary antibodies were used for western blot analysis: TNF-α (1:500, 52B83; sc -52,746, Santa Cruz), total-nuclear factor-kappa B (NF-κB) (1:1000, #8242; Cell signaling Technology, Danvers, MA, USA), phospho-NF-κB (1:1000, #3033; Cell signaling Technology, Danvers, MA, USA), cyclooxygenase 2 (COX2) (1:1000, #4842S; Cell signaling Technology, Danvers, MA, USA), superoxide dismutase 2 (SOD2) (1:1000, 13141S; Cell signaling Technology, Danvers, MA, USA), heme oxygenase 1 (Hmox1) (1:1000, 70081S; Cell signaling Technology, Danvers, MA, USA), glutathione peroxidase (GPX) (1:1000, B-6; sc -133,160, Santa Cruz), Catalase (1:3000, H-9; sc271803, Santa Cruz), and α-tubulin (1:3000, #2144; Cell signaling Technology, Danvers, MA, USA).

### 2.12. Statistical analyses

Data are expressed as means ± standard deviation (SD) or individual values (*n* = 12). The VAS were presented as raw values and as the area under the curve (AUC) during the experimental period. The AUC was calculated as the sum of seven trapezoid areas separated by each measurement time point. Student’s *t*-test was used for comparisons between the two groups. The effect of each supplement or time (day) on the measured results was determined by a two-way analysis of variance (ANOVA) followed by Tukey’s multiple comparisons test. The GraphPad Prism 7 software (GraphPad, Inc., San Diego, CA, USA) was used for all statistical calculations, and the significance level was set to *P* < 0.05 for all cases.

## 3. Results

### 3.1. P*erformance trial (eggbeater kick ability)*

To determine whether MA affects treading water performance, we measured the maximum number of sets of high-intensity interval exercises with an eggbeater kick for water polo athletes. The exhaustion set count for the performance trial was lower than before the intervention but was not affected by MA intake (data are not shown). Lactate concentration levels were elevated after the performance trial, but no effect of MA was observed (data are not shown).

MA intake did not affect performance tests.

### 3.2. Perceptual fatigue and muscle soreness

**Figure 2.**
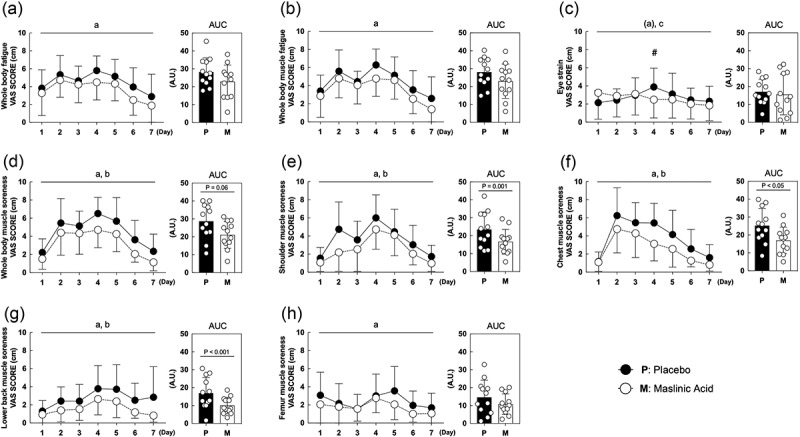
MA intake attenuates perceptual fatigue and muscle soreness during the one-week intervention. (a) whole body fatigue, (b) whole body muscle fatigue, (c) eye strain, (d) whole-body muscle soreness,(e) shoulder muscle soreness, (f) chest muscle soreness, (g) lower back muscle soreness and (h) femur muscle soreness. All data are expressed as the means and individual values (*n* = 12). Significant differences were assessed using a two-way ANOVA followed by Tukey’s multiple comparisons test. Significant differences: a: main effect of MA (*P* < 0.05); (a): main effect of MA (*P* < 0.1); b: main effect of time(*P* < 0.05); c: interaction of MA and time (*P* < 0.05); #: between placebo and MA after intervention *(P* < 0.05). Significant differences in AUC are shown individually.

We measured perceptual fatigue and muscle soreness as the primary outcomes. Regarding fatigue, whole-body fatigue and muscle fatigue were lower in the MA condition compared with those in the placebo condition (Figure 2*A. B*, main effect of MA: *P* < 0.05). Eye strain showed no difference in either condition, but interaction occurred on Day 1 and 4 in the placebo condition (Figure 2*C*, Interaction: *P* < 0.05). The degree of muscle soreness in the whole-body and each body part (shoulder, chest, lower back and femur) were also lower in the MA condition compared with that in the placebo condition (Figure 2*D, E, F, G, H*, main effect of MA: *P* < 0.05). These results indicate that MA attenuates perceptual fatigue and muscle soreness.

### 3.3. Inflammatory and damage markers in blood

**Figure 3.**
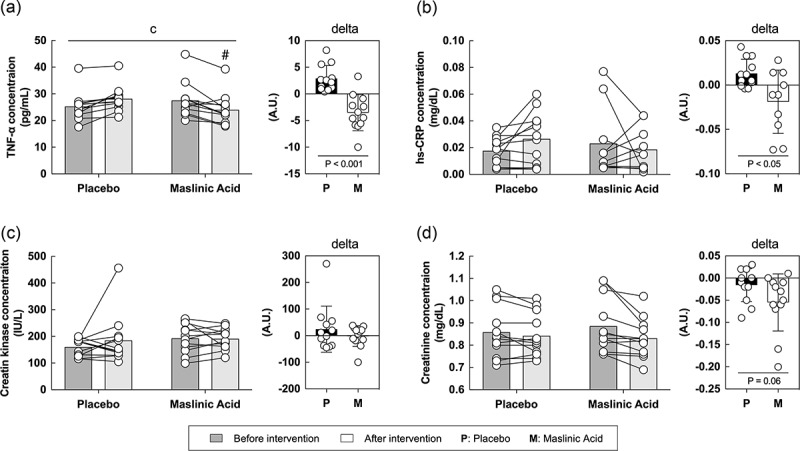
MA intake inhibits the accumulation of inflammation in the blood during a one-week intervention. Levels of (A) TNF-α, (B) hs-CRP, (C) creatin and (D) creatinine concentration. All data are expressed as the means and individual values (*n* = 12). Paired data are connected with lines consistently across all figures where possible. Significant differences were assessed by a two-way ANOVA followed by Tukey’s multiple comparisons test. Significant differences: c: interaction of MA and time (*P* < 0.05); #: between placebo and MA after intervention *(P* < 0.05). Significant differences in the delta are shown individually.

To elucidate the mechanism of the anti-inflammatory effects of MA, we measured inflammatory (TNF-α and high sensitive C-reactive protein) and damage (CK and creatinine) biomarkers in the blood. For TNF-α, an interaction was observed between the placebo and the MA condition after intervention and the rate of change in TNF-α before to after intervention was lower in the MA condition compared with that in the placebo condition (Figure 3*A*, interaction: *P* < 0.05). The hs-CRP, CK, and creatinine levels were not changed during the intervention. However, the rate of change in hs-CRP was significantly lower in the MA condition than in the placebo condition (*P* < 0.05). Although no statistically significant differences were observed (Figure 3*B, C, D*), the rate of change in creatinine, a marker of damage, showed a decreasing trend in MA condition after the intervention (Figure 3*D, P* = 0.06). These results suggest that MA decreased inflammatory and damage markers in the blood.

### 3.4. Oxidative stress (TBARS) in blood

**Figure 4.**
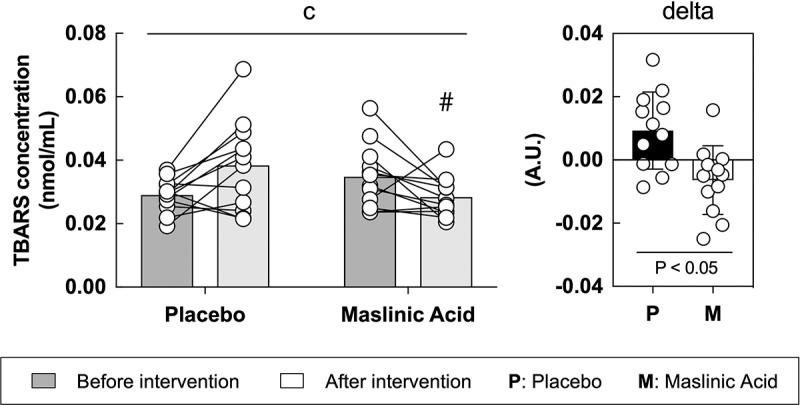
MA intake inhibits oxidative stress accumulation in blood during a one-week intervention. All data are expressed as means and individual values (*n* = 12). Paired data are connected with lines. Significant differences were assessed by a two-way ANOVA followed by Tukey’s multiple comparisons test. Significant differences: c: interaction of MA and time (*P* < 0.05); #: between placebo and MA after intervention *(P* < 0.05). Significant differences in the delta are shown individually.

To determine the effect of MA on oxidative stress, we measured TBARS in the serum. TBARS is a representative oxidative stress marker and is a protein that is primarily produced when fat is oxidized. Between the placebo and MA conditions after the intervention, an interaction was observed (interaction: *P* < 0.05), and the rate of change in TBARS before to after intervention was significantly lower in the MA condition compared with that in the placebo condition (Figure 4, *P* < 0.05).

### 3.5. Inflammatory and antioxidant related proteins in vitro

**Figure 5.**
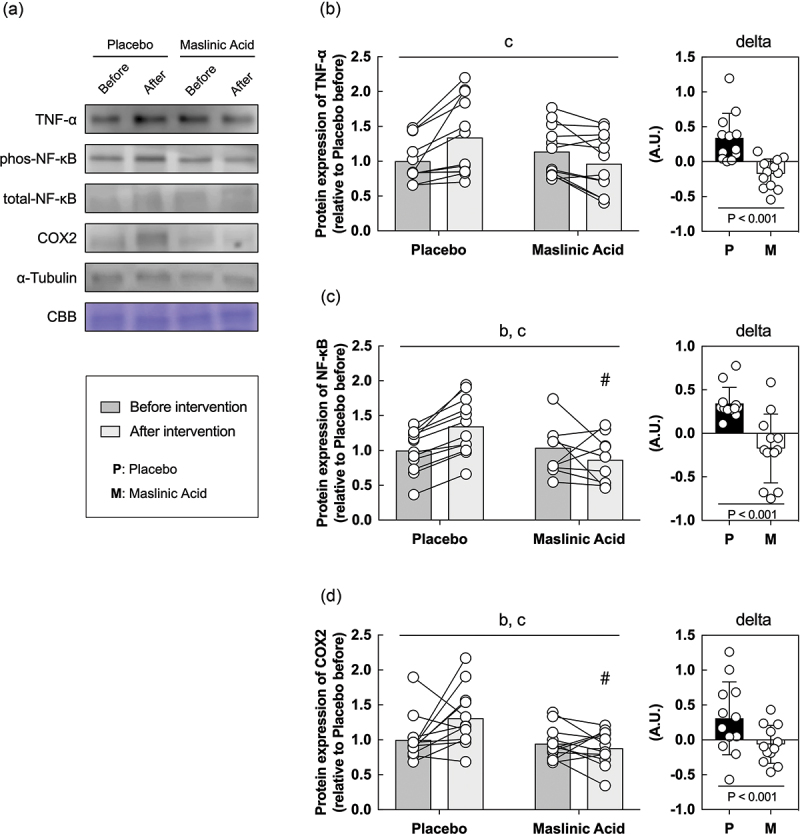
Inflammatory related proteins are decreased within myotubes treated with serum from MA-conditioned participants after intervention. (a) western blot band images, protein expression levels of (b) TNF-α, (c) NF-κB, and (d) COX2. All data are expressed as means and individual values (*n* = 12). Paired data are connected with lines consistently across in all figures. Significant differences were assessed by a two-way ANOVA followed by Tukey’s multiple comparisons test. Significant differences: b: main effect of time (*P* < 0.05); c: interaction of MA and time (*P* < 0.05); #: between placebo and MA after intervention *(P* < 0.05). Significant differences in the delta are shown individually.

**Figure 6.**
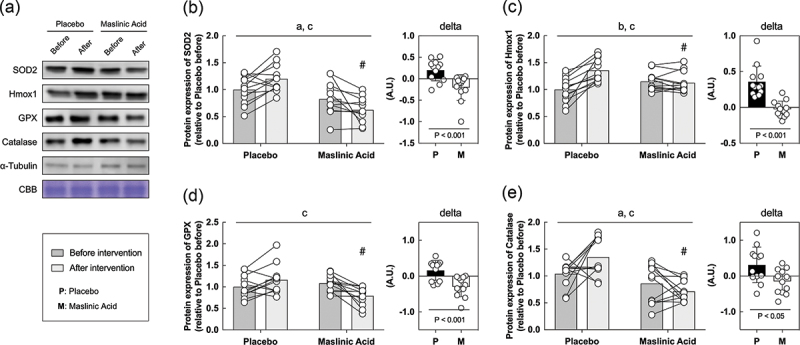
Antioxidant factor-related proteins are also decreased within myotubes treated with serum from MA-conditioned participants after intervention. (a) western blot band images, protein expression levels of (b) SOD2, (c) Hmox1, (d) GPX, and (e) catalase. All data are expressed as means and individual values (*n* = 12). Paired data are connected with lines consistently across in all figures. Significant differences were assessed by a two-way ANOVA followed by Tukey’s multiple comparisons test. Significant differences: a: main effect of MA (*P* < 0.05); b: main effect of time (*P* < 0.05); c: interaction of MA and time (*P* < 0.05); #: between placebo and MA after intervention *(P* < 0.05). Significant differences in the delta are shown individually.

Finally, we performed an *in vitro* study using sera obtained before and after the intervention to determine the effects of MA administration on the body, rather than MA itself. *In vitro* models using serum from athletes who take supplements are useful for understanding the efficacy of supplements and their detailed molecular mechanisms. C2C12 myotubes were starved of serum and amino acids for 1 hour, incubated with conditioned human serum (10%) for 24 h, and collected the cells. Because MA decreased TNF-α and TBARS in serum (Figures 3, 4), we focused on the expression of inflammatory and antioxidant factors. An interaction of TNF-α, NF-κB, and COX2 which are inflammatory regulated proteins is significantly lower with the addition of serum after the intervention than before (Figure 5*A, B, C, D*, interaction: *P* < 0.05). Additionally, an interaction of SOD2, Hmox1, GPX, and catalase, which are responsive to oxidative stress, also significantly lower with the addition of serum after the intervention than before (Figure 6*A, B, C, D, E*, interaction: *P* < 0.05). The rate of change in inflammation and oxidative stress-related protein expression was significantly lower for all markers in MA condition than those in placebo (Figures 5, 6, *P* < 0.05). These results suggest that MA intake did not accumulate inflammatory factors and oxidative stress, and antioxidant factors did not change.

## 4. Discussion

The findings of this study show that seven consecutive days of MA supplementation effectively reduced perceptual fatigue, soreness, inflammation, and oxidative stress markers in the blood in highly trained water polo athletes. In an *in vitro* experiment, we also found that the addition of serum from MA-treated participants to the C2C12 myotubes did not increase oxidative stress. These results indicate that MA intake did not affect the performance of water polo athletes, which is similar to that of previous studies [12,13,15,16]. MA reduced inflammation, and oxidative stress, suggesting that MA might be effective for athletic conditioning.

### 4.1. MA and exercise performance

This is the first study to report the effects of MA supplementation on inflammation, oxidative stress, and swimming (treading water) performance in team sport athletes. Contrary to our (and previous studies) hypothesis, the MA ingestion did not affect performance, but suppressed perceptual fatigue and muscle soreness. These results may be linked to the magnitude of inflammation, oxidative stress and muscle damage induced by training in this study being substantially lower than in previous studies [42,43]. The mechanical strain caused by water polo activity is likely lower than that in endurance sports, such as running or cycling [42,43], because water polo athlete can float and involved in an intermittent style of play. In support of this notion, Nieman et al. suggested that mechanical damage during exercise can significantly alter the magnitude of the inflammatory response [44]. On the other hand, studies on rugby, a contact sport, have reported that inflammatory factors in the blood increase during a rugby match, but only to a small extent [45]. Thus, our participant’s small magnitude of inflammation may have been insufficient to indicate the anti-inflammatory effects resulting from MA supplementation. We examined the rate of change before and after the intervention with and without MA intake, and MA intake reduced inflammation, damage, and oxidative stress marker levels. These results might be due to an anti-inflammatory effect of MA as demonstrated in our previous study [13]. Therefore, our results suggest that the degree of perceptual fatigue and muscle soreness may be associated with the rate of change of muscle damage and inflammation.

### 4.2. Perceptual evaluation

We observed the main effect of MA in all indexes of the evaluation by VAS, which is the main outcome of this study. Our previous study reported that MA attenuates arthralgia pain in the elderly and arthritis in inflammatory mouse models [12,13]. Here, we found a similar effect on skeletal muscle. Although MA was taken after afternoon exercise in the present study, it suppressed perceptual fatigue and muscle soreness as measured by VAS by the following morning. This suggests that MA works not only as immediate inhibitor of inflammation, but also its metabolites also reduce fatigue and muscle soreness for several hours. The timing of MA intake coincided with the onset of exercise-induced inflammation, suggesting that the expression of inflammatory factors in the blood was suppressed. In contrast, muscle damage markers such as CK and myogenin were increased in the blood concentration 24 h after exercise [46]. This is different from the timing of MA release into the blood, and thus may not have been affected. However, serum creatinine after the intervention was lower following MA ingestion, indicating that MA suppresses muscle damage. Future studies will consider the timing of MA ingestion and the timing of muscle damage, which may further reveal the potential benefits of MA.

### 4.3. Evaluation of inflammatory and oxidative stress in blood

We quantified some factors associated with inflammation, damage, and oxidative stress in the blood plasma to determine the effects of MA intake. Reduced inflammation, damage, and oxidative stress after intervention indicate the maintenance of muscular function. It likely mitigates soreness, possibly explaining the preservation and recovery of strength observed in previous studies in which MA was consumed [47]. The present study revealed that TNF-α and hs-CRP, (major inflammatory markers), and TBARS (oxidative stress marker) decreased significantly after the intervention. The rate of change was also significantly lower in the MA condition than in the placebo condition. Creatinine (muscle damage marker) also showed a decreasing trend in the MA condition after intervention. In the current study, the inflammation marker TNF-α was significantly lower in the MA condition than in the placebo condition. Previous studies have shown that MA suppresses inflammation in blood and tissues (6, 7), and the present study confirmed similar results, indicating the potent anti-inflammatory effect of MA. Although the participants in this experiment routinely performed high-intensity exercise and therefore had a relatively low inflammatory response in their blood. However, the main effect of MA was confirmed, suggesting that it may be an effective supplement for highly trained athletes as well. Because of the limitations of the current intervention, we did not apply any damage-inducing exercise or contraction modalities. Future studies should examine different exercise modalities, durations, muscle damage and inflammatory responses between activities to determine the effects of MA on damage markers that vary with exercise modality, such as CK.

### 4.4. Effects of MA-ingested serum on cultured cells

As a new insight, we investigated whether the addition of serum before and after the intervention from the participants to cultured cells would affect the protein expression on inflammation or antioxidant. This experiment enables us to examine in more detail the effects of MA and metabolites resulting from MA intake. Although our study here did not allow us to examine the effects of biopsy on protein expression in skeletal muscle, we were able to use the previously reported in vitro model of Allen et al. to understand the mechanisms that inhibit inflammation and oxidative stress accumulation in MA from blood and in vitro data [41]. This approach not only minimizes invasive damage to active athletes, but also provides a detailed understanding of the mechanisms by which MA suppresses exercise-induced inflammation and oxidative stress. In the present study, muscle cells, to which serum from MA-treated participants was added, exhibited a lower response to inflammation and oxidative stress than those from placebo-treated participants. Thus, daily MA intake did not cause accumulate inflammatory factor and oxidative stress, and therefore, inflammatory, and antioxidant-related proteins were not expressed. The placebo serum used in this study increased TNF-α, a marker of inflammatory, and TBARS, a marker of oxidative stress (Figures 4, 5), indicating that adding this serum to cells increased inflammatory or antioxidant-related proteins in response to inflammatory or oxidative stress. It is likely that the addition of serum from MA-treated participants did not increase inflammatory or antioxidant-related proteins in the cells. These data indicate that the addition of human serum to the cell culture medium is an effective tool for studying how MA reduces inflammatory and oxidative stress. However, it is worth noting that there are limitations to this approach. Myotubes were conditioned with only 10% human serum to maintain cell viability in the present study. Although this induced some changes in antioxidant markers, this low concentration may not maximally induce changes in oxidative stress. We did not prepare human primary skeletal muscle cells, but used C2C12 murine cells, which may cause issues related to cross-species differences. Therefore, future studies should aim to replicate this work using human primary skeletal muscle cells. A strength of this study is that it was conducted on elite water polo athletes. This is an extremely rare study, so the potential effects of MA may be examined extensively in other athletes, especially in runners or cyclists with high metabolic variability, and in competitive athletics such as rugby or judo, where injury and inflammation should be very high.

## 5. Limitations

One limitation of this study was the short duration of MA ingestion. Our study was conducted for a short period of one week to ascertain the impact of MA on inflammation and injury. In addition, because this study was conducted with active athletes, it was limited to one week because of the constraints of accommodating their schedules. Therefore, future studies will need to extend the duration of the study to examine the effects of MA in more detail. Additionally, since we did not measure training load during intervention, it is possible that differences in training load may have affected the degree of damage/inflammation. Furthermore, the positional qualities of water polo players may have affected the training load, therefore future studies should include individual differences in protocols. Another limitation is that the participants of the experiment were rare top water polo athletes in Japan. By targeting athletes with well-defined athlete and competition characteristics, such as power and endurance athletes, rather than complex water polo athletes, it may be possible to confirm the detailed effects of MA on metabolic characteristics and to inform nutritional strategies according to energy mechanisms. Future research should consider the degree of competition, gender differences, and the competition itself in order to generalize the outcomes of this study and make them more applicable to the field.

## 6. Conclusion

In this study, we determined the effect of MA on fatigue and muscle soreness in a randomized, double-blind, cross-over, and placebo-controlled trial. Our results suggest that MA intake for one-week significantly attenuated fatigue and muscle soreness by reducing inflammation and damage. Furthermore, we found novel MA benefits, such as the inhibition of oxidative stress in skeletal muscle cells by adding of serum from MA-treated athletes using an *in vitro* model. Therefore, supplementation with MA may be a useful strategy for attenuating fatigue and muscle soreness, especially for athletes participating in strenuous sports, such as water polo athletes.

## Abbreviations

ANOVA: analysis of variance, AUC: area under the curve, BMI: Body mass index, CK: Creatine kinase, COX2: Cyclooxygenase 2, DMEM: Dulbecco’s modified Eagle’s medium, GPX: Glutathione peroxidase, Hmox1: Heme oxygenase 1, hs-CRP: High-sensitivity C-reactive protein, MA: maslinic acid, mTOR: Mechanistic target of rapamycin, NF-κB: Nuclear factor-kappa B, SD: Standard deviation, SOD2: Superoxide dismutase 2, TBARS: Thiobarbituric acid reactive substance, TNF-α, Tumor necrosis factor α, VAS: Visual analog scale.

## Data Availability

The sharing of data in an open-access repository was not included in our participants consent. Thus, in accordance with standard ethical practice, data may only be available on request from the corresponding author.
